# Segmental Colitis Associated With Diverticulosis

**DOI:** 10.7759/cureus.38724

**Published:** 2023-05-08

**Authors:** Om V Sakhalkar, Arnav Goyal, Abdul Rahman Abualruz

**Affiliations:** 1 Radiology, Augusta University Medical College of Georgia, Augusta, USA

**Keywords:** chronic colitis, diverticulosis complications, sigmoid diverticulosis, sigmoid diverticulitis, segmental colitis

## Abstract

Segmental colitis associated with diverticulosis (SCAD) is a rare entity characterized by segmental circumferential colonic wall thickening involving the sigmoid and/or left colon in the presence of colonic diverticulosis. We present the case of a 57-year-old female with a past medical history of colonic diverticulosis who presented with chronic intermittent abdominal pain, non-bloody diarrhea, and hematochezia. Imaging revealed long-segment circumferential colonic wall thickening involving the sigmoid and distal descending colon with engorged vasa recta without significant inflammation around the colon or diverticula, consistent with SCAD. Colonoscopy showed diffuse mucosal edema and hyperemia of the descending and sigmoid colon with easy friability and erosions primarily affecting the inter-diverticular colonic mucosa. Pathology showed changes of chronic colitis including inflammation in the lamina propria, crypt distortion, and granuloma formation. Treatment with antibiotics and mesalamine was initiated with improvement in symptoms. This case highlights the importance of considering segmental colitis associated with diverticulosis in patients with chronic lower abdominal pain and diarrhea in the setting of colonic diverticulosis, and the need for a thorough workup including imaging, colonoscopy, and histopathology to differentiate it from other types of colitis.

## Introduction

Colonic diverticulosis is a common condition that affects up to 65% of individuals over the age of 65 in the United States [1]. Colonic diverticulosis may be asymptomatic or symptomatic. Diverticular disease is defined as clinically significant and symptomatic diverticulosis due to diverticular bleeding, diverticulitis, segmental colitis associated with diverticulosis (SCAD), or symptomatic uncomplicated diverticular disease. SCAD is characterized by segmental circumferential thickening of the colonic wall that contains diverticulosis, along with engorgement of mesenteric vessels and mild pericolonic inflammation not centered around colonic diverticula. Patients with SCAD typically present with lower abdominal pain, chronic diarrhea, and intermittent hematochezia. While the prevalence of SCAD is relatively low, at 1.5%, it is more common in males and older adults [2]. Diagnosis of SCAD can be challenging due to its resemblance to other types of colitis, particularly inflammatory bowel disease. CT is the imaging modality of choice for SCAD and typically demonstrates long-segment circumferential colonic wall thickening involving the sigmoid and/or left colon in the region of colonic diverticulosis with engorged mesenteric vessels and mild pericolonic inflammation not centered around colonic diverticula.

## Case presentation

**Figure 1:**
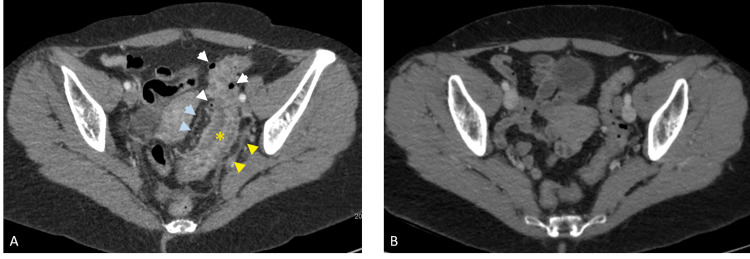
Abdominal CT at the time of patient’s presentation and after management (A) The axial CT image shows segmental sigmoid colon wall thickening (yellow asterisk), mild pericolonic inflammation (yellow arrowheads) sparing colonic diverticula (white arrowheads), and engorged vasa recta (blue arrowheads). (B) The axial contrast-enhanced CT image obtained after management shows resolution of sigmoid colon wall thickening and surrounding inflammation.

**Figure 2:**
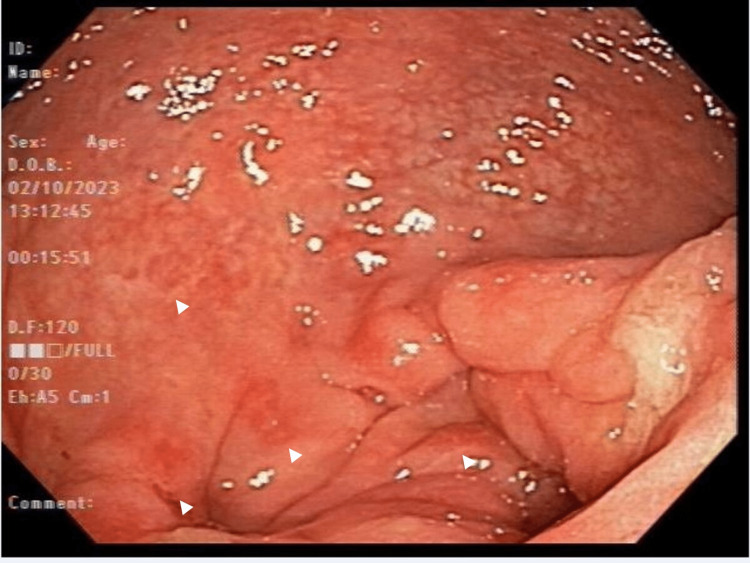
Colonoscopy demonstrating mucosal edema and hyperemia of the sigmoid colon with easy friability and erosions (white arrowheads) primarily affecting the inter-diverticular colonic mucosa

A 57-year-old White female presented to the emergency department with chronic intermittent abdominal pain, non-bloody diarrhea, and hematochezia. Her pain was mostly localized in the left lower quadrant and partly relieved by a bowel motion. There was no constitutional symptom or history of weight loss. She had a past medical history of hemorrhoids and colonic diverticulosis, with no significant past surgical history. Vital signs were within normal limits. On physical examination, the abdomen was soft and tender to deep palpation in the left lower quadrant. There was no rebound tenderness or guarding. The examination was otherwise unremarkable. Complete blood count showed normal hemoglobin at 13 g/dl and WBC 7200/mm^3^. A CT scan of the abdomen and pelvis was performed and showed a long segment of circumferential colonic wall thickening involving the sigmoid and distal descending colon in the region of colonic diverticulosis with engorged vasa recta and mild pericolonic inflammation not surrounding sigmoid diverticula suggestive of segmental sigmoid colitis rather than diverticulitis (Figure 1). Fecal calprotectin and lactoferrin levels were elevated and stool culture was negative for an infectious etiology. Colonoscopy showed diffuse mucosal edema and hyperemia of the descending and sigmoid colon with easy friability and erosions primarily affecting the inter-diverticular colonic mucosa (Figure 2). The remainder of the colon and the terminal ileum were normal. Tissue samples were obtained and histopathology revealed features of chronic colitis including crypt distortion and inflammation in lamina propria with granuloma formation.

## Discussion

Patients who have colonic diverticulosis may develop segmental colitis, which is most commonly found in the sigmoid colon [1]. On imaging, segmental circumferential thickening of the colonic wall that contains diverticulosis (usually in the sigmoid colon and/or left colon) along with engorgement of mesenteric vessels is the characteristic feature of segmental colitis associated with diverticulosis. CT is the imaging modality of choice and typically demonstrates long-segment circumferential colonic wall thickening involving the sigmoid and/or left colon in the region of colonic diverticulosis with engorged mesenteric vessels without significant inflammation around the colon or colonic diverticula. SCAD spares the rectum and terminal ileum that helps to differentiate it from inflammatory colitis on imaging. However, clinical symptoms, colonoscopy findings, and histopathological features of SCAD may resemble other types of colitis, particularly inflammatory colitis [1-4]. Inflammatory bowel disease and SCAD share similar clinical presentations, making them difficult to distinguish based on clinical symptoms alone. However, imaging, colonoscopy, and histopathology can help differentiate SCAD from inflammatory bowel disease [5].

Typically, SCAD involves the sigmoid colon with or without the left colon, where colonic diverticulosis is often present. On the other hand, ulcerative colitis typically involves the rectum and may extend continuously through the colon, while Crohn's disease can affect the terminal ileum and may involve locations beyond those associated with diverticulosis. In contrast, SCAD spares the rectum and right colon/terminal ileum. The differential diagnosis for SCAD also includes several conditions. Diverticulitis can have a similar clinical presentation to SCAD, but it usually presents acutely. On CT, acute diverticulitis is characterized by colonic wall thickening with pericolic inflammation centered around an inflamed diverticulum that is more severe than the degree of colonic wall thickening seen in SCAD. On colonoscopy, diverticulitis typically affects the diverticular orifices and the surrounding mucosa, while sparing the interdiverticular mucosa [6]. Infectious colitis is characterized by an acute presentation of abdominal pain, diarrhea, and fever. On imaging, colonic wall thickening is usually more pronounced than the surrounding inflammation. Stool cultures typically show positive results for the underlying organism. Ischemic colitis can manifest with segmental colonic wall thickening that affects the watershed areas, including the splenic flexure (Griffiths point) and rectosigmoid junction (Sudeck's point), on imaging. Additional findings may vary depending on the acuity and etiology of the ischemia, which can include arterial thromboembolism, venous thrombosis, and hypoperfusion. There are no established guidelines for the management of SCAD, potentially due to its low prevalence [7,8]. The initial treatment course of SCAD utilizes antibiotics (ciprofloxacin and metronidazole). If patients fail to respond to antibiotics, mesalamine or corticosteroids are suggested. Surgical resection is preserved for severe cases with intractable symptoms not responding to medical therapy [7]. Limited data is available on the role of immunomodulators and biological agents in the management of SCAD [7,8].

## Conclusions

SCAD should be considered in patients with chronic lower abdominal pain and diarrhea who have colonic diverticulosis. Imaging, colonoscopy, and histopathology are necessary to differentiate SCAD from other types of colitis. Treatment with antibiotics and mesalamine may be effective in improving symptoms.
